# Suppression of apoptosis inhibitor c-FLIP selectively eliminates breast cancer stem cell activity in response to the anti-cancer agent, TRAIL

**DOI:** 10.1186/bcr2945

**Published:** 2011-09-14

**Authors:** Luke Piggott, Nader Omidvar, Salvador Martí Pérez, Matthias Eberl, Richard WE Clarkson

**Affiliations:** 1 Life Sciences Building, Cardiff University School of Biosciences, Museum Avenue, Cardiff, CF10 3AX, Wales, UK; 2 Department of Infection, Immunity and Biochemistry, Cardiff University School of Medicine, Heath Park Campus, Cardiff, CF14 4XN, Wales, UK

## Abstract

**Introduction:**

It is postulated that breast cancer stem cells (bCSCs) mediate disease recurrence and drive formation of distant metastases - the principal cause of mortality in breast cancer patients. Therapeutic targeting of bCSCs, however, is hampered by their heterogeneity and resistance to existing therapeutics. In order to identify strategies to selectively remove bCSCs from breast cancers, irrespective of their clinical subtype, we sought an apoptosis mechanism that would target bCSCs yet would not kill normal cells. Suppression of the apoptosis inhibitor cellular FLICE-Like Inhibitory Protein (c-FLIP) partially sensitizes breast cancer cells to the anti-cancer agent Tumour Necrosis Factor-Related Apoptosis Inducing Ligand (TRAIL). Here we demonstrate in breast cancer cell lines that bCSCs are exquisitely sensitive to the de-repression of this pro-apoptotic pathway, resulting in a dramatic reduction in experimental metastases and the loss of bCSC self-renewal.

**Methods:**

Suppression c-FLIP was performed by siRNA (FLIPi) in four breast cancer cell lines and by conditional gene-knockout in murine mammary glands. Sensitivity of these cells to TRAIL was determined by complementary cell apoptosis assays, including a novel heterotypic cell assay, while tumour-initiating potential of cancer stem cell subpopulations was determined by mammosphere cultures, aldefluor assay and *in vivo *transplantation.

**Results:**

Genetic suppression of c-FLIP resulted in the partial sensitization of TRAIL-resistant cancer lines to the pro-apoptotic effects of TRAIL, irrespective of their cellular phenotype, yet normal mammary epithelial cells remained refractory to killing. While 10% to 30% of the cancer cell populations remained viable after TRAIL/FLIPi treatment, subsequent mammosphere and aldefluor assays demonstrated that this pro-apoptotic stimulus selectively targeted the functional bCSC pool, eliminating stem cell renewal. This culminated in an 80% reduction in primary tumours and a 98% reduction in metastases following transplantation. The recurrence of residual tumour initiating capacity was consistent with the observation that post-treated adherent cultures re-acquired bCSC-like properties *in vitro*. Importantly however this recurrent bCSC activity was attenuated following repeated TRAIL/FLIPi treatment.

**Conclusions:**

We describe an apoptotic mechanism that selectively and repeatedly removes bCSC activity from breast cancer cell lines and suggest that a combined TRAIL/FLIPi therapy could prevent metastatic disease progression in a broad range of breast cancer subtypes.

## Introduction

Recognition that breast cancer is a heterogeneous disease has helped shape advances in therapy, leading to more targeted therapeutic strategies and improved survival rates in discrete disease subgroups [1]. This is exemplified by the advent of therapeutic agents targeting estrogen-receptor positive (ER^+^) and HER2-positive (HER2^+^) breast cancers, which make up approximately 70% of all breast tumours [2,3]. Despite these improvements, however, tumours often relapse due to innate or acquired resistance to the therapeutic insult. At the centre of this problem lies additional tumour heterogeneity whereby a small population of cells within, or possibly outside, the tumour are both resistant to drugs and provide the source of new tumour growth [4,5]. These cells also contribute directly to the seeding of secondary tumours at distal sites, the primary cause of mortality in breast cancer patients [6]. These drug resistant cancer initiating cells, often referred to as breast Cancer Stem Cells (bCSCs), have been demonstrated functionally for both human and mouse mammary tumours and tumour cell lines [7-13]. Experiments on human breast tumours in mouse models, for example, indicate that when these cells were deleted, the remaining cells were unable to sustain new tumour growth [11,13,14]. There is, therefore, considerable interest in targeting CSCs within tumours with cytotoxic agents as a cure for breast and other cancers and where possible to broaden the specificity of therapeutic agents to treat as wide a patient group as possible.

Tumour Necrosis Factor (TNF)-Related Apoptosis Inducing Ligand (TRAIL) is a promising anticancer agent that exhibits tumour specificity with only mild side effects observed in clinical trials for the treatment of colorectal cancer, non-small cell lung carcinoma and non-Hodgkins lymphoma [15,16]. In breast cancer, however, its therapeutic potential is limited by the fact that the majority of breast cancer cell types are resistant to TRAIL [17,18]. This has prompted much interest in identifying agents that might increase TRAIL sensitivity in a larger cohort of breast cancer patients. Moreover, stem cells, including cancer stem cells, are documented to be resistant to TRAIL [16,19,20], suggesting that without further sensitization of the tumour-initiating cell sub-population, patients are likely to relapse following TRAIL therapy.

TRAIL targets tumour cells for instructive cell death via the cell-surface receptors TRAIL-R1 (DR4) and TRAIL-R2 (DR5), which initiate the formation of death inducing signalling complexes (DISCs) ultimately leading to the activation of the caspase cascade [21]. A number of studies have described agents that sensitize one or more breast cancer subtypes to TRAIL, the majority of which implicate components of the apoptosis regulatory machinery as the underlying causes of sensitization [22-35]. Common to a number of these studies is the observation that the endogenous inhibitor of death receptor killing, cellular FLICE-Like Inhibitory Protein (c-FLIP), is down-regulated during the sensitization process [22,26,28,30-32,34]. c-FLIP is a non-redundant antagonist of caspases -8 and -10, preventing these caspases from binding to the DISC and thus inhibiting autolytic cleavage and subsequent activation of downstream executioner caspases [36] following stimulation by TRAIL. The suppression of c-FLIP has been shown to sensitize some breast cancer cell lines to TRAIL mediated killing, raising the possibility that such a mechanism could be targeted in breast cancer patients [22,31,32,37-39]. However, several questions concerning the specificity of c-FLIP in breast cancer remain that would significantly impact on its prospects as a therapy for breast cancer. These include: whether suppressing c-FLIP in non-tumour cells compromises their viability; whether a broad range of breast cancer subtypes are affected by c-FLIP sensitization; and of particular clinical significance, whether the normally chemo-resistant CSC sub-populations within each of these heterogeneous subtypes are sensitive to de-repression of this apoptotic pathway.

Here we addressed each of these clinically relevant questions by selectively targeting c-FLIP in pre-clinical models of breast cancer. We looked at the effects of suppressing c-FLIP in non-tumourgenic cells, and showed that c-FLIP exhibited tumour cell specificity, similar to that previously ascribed to TRAIL in other tumour types [15,40,41]. Moreover, we demonstrated that the de-repression of TRAIL by c-FLIP inhibition selectively eliminated breast cancer stem cells (bCSCs) from tumour cell populations, irrespective of their HER2/ER receptor status and despite CSC plasticity within the surviving tumour cell population. These observations were then confirmed in *in vivo *models of breast cancer whereby primary tumourgenesis was reduced by 80% and the seeding of new tumour growth at distal sites, leading to metastatic disease, was almost completely inhibited. These findings demonstrate potent cellular responses to TRAIL sensitization that have important clinical implications for the advent of new therapeutic strategies for breast cancer patients.

## Materials and methods

All experiments were performed with the approval of the University of Cardiff School of Biosciences Ethics Committee and animal work was performed in accordance with the Home Office Animals (Scientific Procedures) Act 1986 under project licence 30/2849.

### Cell culture

Four human breast cancer cell lines BT474^ER+HER2+^, SKBR3^ER-HER2+^, MCF-7 ^ER+HER2-^, MDA-MB-231^ER-HER2-^; a murine mammary tumour cell line, N202.1A (from P-L Lollini, Sezione di Cancerologia, Bologna, Italy); the non-tumourigenic cell lines human MCF10A (from T. Stein, University of Glasgow, UK) and murine EPH4 (from C. Watson, University of Cambridge, UK) were maintained in DMEM (MDA-MB-231, EPH4), or RPMI 1640 medium (SKBR3, MCF-7 and BT-474), supplemented with 10% foetal bovine serum, 1% penicillin-streptomycin and 0.5% L-glutamine at 37°C in 5% CO_2_. Monolayer MCF10A cells were cultured in DMEM/F12, 5% horse serum, 20 ng/ml EGF, 0.5 mg/ml hydrocortisone, 100 ng/ml cholera toxin, 10 μg/ml insulin.

### siRNA

Small interfering RNAs (siRNA) targeting two unique sequences in human c-FLIP (FLIPi - Sense: GGAUAAAUCUGAUGUGUCCUCAUUA, Anti-Sense: UAAUGAGGACACAUCAGAUUUAUCC) and a non-specific scrambled control (SCi - Sense: GGACUAAUAGUUGUGCUCCAAUUUA, Anti-Sense: UAAAUUGGAGCACAACUAUUAGUCC) RNA were used in reverse transfections (Invitrogen Life Technologies Ltd, Paisley, UK). Cells were trypsinised and resuspended at a density of 1 × 10^5 ^cells/ml and seeded into wells containing 20 μl of 100 nM siRNA in serum free Optimem (Invitrogen Life Technologies Ltd, Paisley, UK)) in a volume of 100 μl per well together with 0.3 μl of Lipofectamine (Invitrogen Life Technologies Ltd). Cells were cultured in the presence of siRNA for 48 hours (MCF-7, MCF10A, EPH4, N202.1A and MDA-MB-231) or 72 hours (SKBR3 and BT474) prior to subsequent assay.

### TRAIL treatment of target cells

Cells were treated with soluble human recombinant TRAIL (SuperKillerTRAIL, Enzo Life Sciences, Exeter, UK) at a concentration of 20 ng/ml for 18 hours at 37°C in 5% CO_2_. For mouse target cells, soluble mouse recombinant TRAIL (Enzo Life Sciences) was added at a concentration of 100 ng/ml for 18 hours.

### Western blot assays

Western blots of cell lysates were performed using the following antibodies: cFLIP (Enzo Life Sciences, NP6, ALX-8040428), ER α (Santa Cruz Biotechnology, Santa Cruz, CA, USA, sc-7207), ErbB2 (Abcam, Cambridge, UK, ab2428), Tubulin (Abcam, ab6160).

### *In vitro *caspase inhibition

Functional blocking of caspases was assessed by co-incubation of cells with siRNA and the caspase inhibitors IETD (1 μM), LEHD (10 μM) and AEVD (10 μM) (R&D Systems, Abingdon, Oxford, UK) to inhibit caspases 8, 9 and 10 respectively. After 48 to 72 hours co-incubation, cells were analysed using Annexin-V APC apoptosis assay (eBioscience Ltd, Hatfield, UK).

### Cell viability and cell death assays

*In heterotypic cell culture assays: *siRNA treated cells were treated with 0.25% trypsin for 10 minutes, washed and stained with PKH67 or PKH26 (Sigma-Aldrich, Gillingham, Dorset, UK). PKH67+ve FLIPi cells and PKH26+ve SCi cells were mixed 1:1 and cultured overnight with or without TRAIL and subsequently resuspended in 4 μl of 1:10 fixable near-IR live/dead stain (Invitrogen) and incubated for 15 minutes at 4°C. Cells were then gated for PKH staining versus live/dead staining using a FACS Canto (Becton Dickinson, Oxford, UK). For detailed protocol, see supplementary data. *In homotypic cell culture assays*: CellTiter blue cell viability assay (Promega UK Ltd, Southampton, UK) and Caspase-Glo assay (Promega) were performed according to the manufacturer's instructions and fluorescence/absorbance/luminescence was assessed using a FluoStar Optima plate reader, while annexin-V APC labelled cells (eBioscience) were analysed by FACS Canto.

### Mouse mammary gland tissue histology and primary culture

All procedures were performed in accordance with the Animals (Scientific Procedures) Act 1986 and approved by the UK Home Office. c-FLIP^fl/fl ^mice [42] were crossed with blg-Cre animals [43] to conditionally delete c-FLIP from mammary epithelium. Mammary tissues from 12-week old and 14-day pregnant blg-Cre/c-FLIP^fl/fl ^females and blg-Cre/c-FLIP^+/+ ^littermate controls were harvested and fixed in 4% paraformaldehyde/PBS (pH 7.4) overnight, and embedded in paraffin. Paraffin sections (5 μm) were placed on slides, de-waxed and stained with H&E. For primary cell culture, mid-pregnant animals were sacrificed and abdominal mammary glands excised and washed in 70% ethanol. Lymph nodes were removed and finely minced tissue was then processed as described [44]. Primary cells were maintained in 5% CO_2_, 5% O_2 _at 37°C.

### Mammosphere culture

Cell lines were dissociated into single cell suspensions and plated in ultra-low attachment plates (Corning Life Sciences, Amsterdam, Netherlands) at a density of 20,000 cells/ml in a serum-free epithelial growth medium (MEBM, Lonza Walkersville, MD, USA), supplemented with B27 (Invitrogen), 20 ng/ml EGF (Sigma-Aldrich), Insulin (Sigma-Aldrich), β-mercaptoethanol and hydrocortisone. After seven days mammospheres were collected by gentle centrifugation (1,100 rpm), dissociated in 0.05% trypsin, 0.25% EDTA and re-seeded at 10,000 cells/ml for subsequent passages.

### Aldefluor (ALDH1) assay

Surviving cell populations were harvested in 0.25% trypsin and collected by gentle centrifugation (1,100 rpm). Cell pellets were then washed twice in PBS prior to Aldefluor assay (Stem Cell Technologies, Grenobles, France) as previously described [45].

### Mouse tumourigenicity assays

*In vivo *tumour initiating capability of siRNA treated cells was assessed by orthotopic mammary fat pad transplantation and tail vein injections of BT474 and MDA-MB-231 cell lines, respectively. BT474 siRNA treated cells were harvested using 1 mM EDTA, washed and resuspended at a density of 5 × 10^6 ^cells/ml in serum-free L15 media. A 1.5 mg, 60-day slow release 17-β estradiol pellet (Innovative Research of America, Sarasota FL, USA) was inserted subcutaneously above the right scapula of anaesthetised athymic nude mice. A total of 1 × 10^6 ^cells were orthotopically injected directly into the abdominal mammary fat pad, with or without 100 ng/ml TRAIL. Mice were then monitored, and when palpable, tumour volume measured twice weekly. MDA-MB-231 cells treated with siRNA were harvested and prepared for injection in the same manner as BT474 cells. Cells were then injected into the mouse tail vein, with or without TRAIL in a volume of 200 μl and mice were sacrificed six weeks post-injection.

### Statistical methods

Throughout the article, data are represented as mean +/- standard error taken over a minimum of three independent experiments, unless otherwise stated. Statistical significance was measured using parametric testing, assuming equal variance, in the majority of experiments with standard t tests for two-paired samples used to assess difference between means.

## Results

### c-FLIP deficiency exhibits tumour cell specificity in mammary epithelium

**Figure 1:**
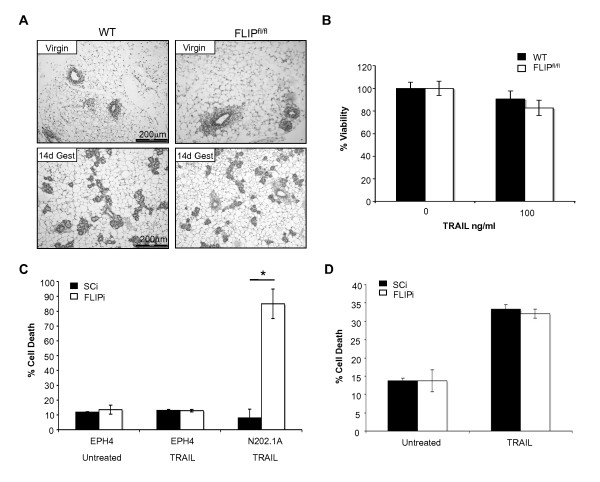
**FLICE-Like Inhibitory Protein (c-FLIP) suppression does not induce cell death in non-tumourigenic mammary cells**. **A**, Haematoxylin/eosin-stained sections of mouse mammary glands excised from virgin or 14-day gestating β-lactoglobulin(BLG)-Cre/c-FLIP^+/+ ^or BLG-Cre/c-FLIP^fl/fl ^animals. Pictures are representative of four animals per group. **B**, Mammary epithelial cells isolated from BLG-Cre/c-FLIP^fl/fl ^(Flip-floxed) and c-FLIP^+/+ ^(WT) animals were grown in primary cell culture and incubated in the presence or absence of 100 ng/ml of soluble (mouse) Tumour Necrosis Factor-Related Apoptosis Inducing Ligand (TRAIL) for 18 hours. Cell viability was analysed using cellTiter blue viability assay. **C**, EPH4 (non-tumourgenic) and N202.1A (tumourgenic) cells were transfected with FLIP siRNA (FLIPi) or control siRNA (SCi) for 48 hours, media was then removed and fresh media with or without 100 ng/ml of mouse TRAIL was added for 18 hours and cell death assessed by flow cytometry as described in methods. **D**, MCF10A (non-tumourgenic) cells were treated and assayed according to C using 20 ng/ml human TRAIL.

It has been reported that TRAIL preferentially targets tumour cells over normal cells [15,40,41]. To determine if the targeted inhibition of c-FLIP exhibited similar specificity for tumour cells, mammary epithelial cell viability was assessed in non-tumourgenic c-FLIP-deficient mouse mammary glands, transformed murine cell lines and in the human breast cell line MCF-10A. c-FLIP was conditionally deleted from mammary epithelial cells of juvenile mice by crossing the blg-Cre transgene [43] into the c-FLIP^fl/fl ^line [42], and the mammary epithelial compartment subsequently assessed in adult virgin and pregnant animals. Mammary epithelial morphogenesis and cell number in blg-Cre/c-FLIP^fl/fl ^mammary glands was indistinguishable from wild-type controls, while isolated primary epithelial cells from both genetic backgrounds exhibited comparable cell viability either in the presence or absence of TRAIL *in vitro *(Figure 1A, B). Furthermore, inhibition of c-FLIP (FLIPi) using murine specific siRNA had no effect on a non-tumourgenic murine cell line's response to TRAIL but significantly reduced viability in a tumourgenic line (Figure 1C). Similarly, in the human non-tumourgenic breast cell line, MCF-10A, cell viability was unaffected by c-FLIP inhibition (FLIPi) alone; however, combined treatment with TRAIL induced a significant cell death response (Figure 1D), confirming previous reports of TRAIL sensitivity in human transformed cell lines [27]. These data indicate that the targeted inhibition of c-FLIP exhibited tumour specific effects, similar to those observed with TRAIL in other cancer types.

### Suppression of c-FLIP (FLIPi) sensitized breast cancer cell lines irrespective of hormone receptor status

Whilst most breast cancers are resistant to TRAIL induced apoptosis, it has recently been reported that mesenchymal breast cancer cell lines that lack hormone receptors (HER2 and ERα) respond to TRAIL treatment [18]. This is a clinically important subgroup of breast cancer, yet it represents only 20 to 25% of the breast cancer patient population. In order to establish the extent to which c-FLIP might broaden the specificity of TRAIL-induced cytotoxicity, we wanted to directly compare the relative sensitivity of different breast cancer subtypes to the combined effects of c-FLIP inhibition and TRAIL treatment.

**Figure 2:**
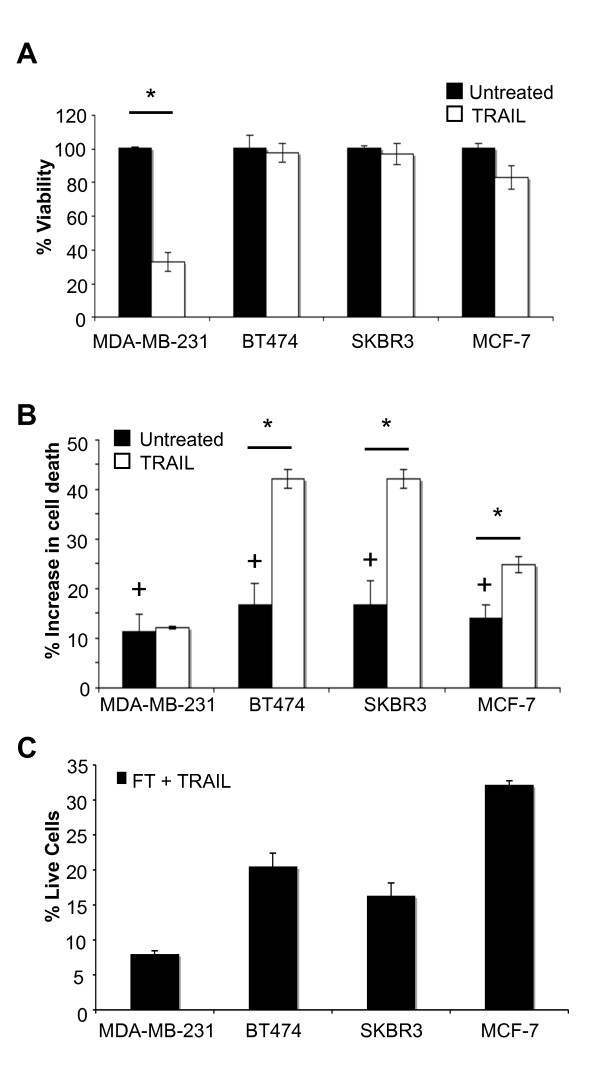
**Suppression of c-FLIP sensitises breast cancer cell lines to TNF-Related Apoptosis Inducing Ligand (TRAIL)**. **A**, Cell lines were incubated with 20 ng/ml soluble TRAIL for 18 hours and cell viability assessed by cellTiter Blue viability assay. Viability is shown as a percentage of the untreated controls for each cell line (*: *P *< 0.01, *n *= 3). **B**, PKH67-stained cells pre-treated with c-FLIP siRNA and PKH26-stained cells pre-treated with control siRNA were mixed and co-cultured for 18 hours in complete media +/-20 ng/ml TRAIL, and assessed by flow cytometry for live/dead cells (see Supplementary figure S2). The percentage increase in cell death of cFLIP siRNA cells compared to their scrambled siRNA controls were plotted for each of the cell lines. TRAIL-treated co-cultures (white bars) and TRAIL-untreated co-cultures (Black bars) were plotted separately. Each co-culture was repeated in three independent experiments. + = *P *< 0.01, for percentage increase in cell death between c-FLIP siRNA and scrambled siRNA control. * = *P *< 0.01, for difference in c-FLIP siRNA mediated death between TRAIL-treated and untreated co-cultures. **C**, Cells were treated as in B and gated to give the percentage of remaining live cells after treatment. FT = c-FLIP siRNA.

We selected four breast cancer cell lines representing all combinations of ERα and HER2 expression: the luminal-like cells BT474^ER+HER2+^, SKBR3^ER-HER2+ ^and MCF-7 ^ER+HER2-^, which represent the majority of breast cancers and the basal-like cell line MDA-MB-231^ER-HER2- ^[46]. Having confirmed their receptor status (Additional file 1 Figure S1) and TRAIL sensitivity in 2D-adherent cell culture (Figure 2A), the effect of inhibiting c-FLIP expression (FLIPi) on cell viability was tested in each cell line using a novel fluorescent heterotypic cell culture assay (Additional file 1 Figure S2). Both c-FLIP_S _and cFLIP_L _transcripts were inhibited by siRNA, resulting in a greater than 70% decrease in expression of c-FLIP in all cell lines (Additional file 1 Figure S3). The suppression of c-FLIP, which had no effect on DR4 or DR5 expression (Additional file 1 Figure S3C), significantly decreased cell viability by 10 to 15% in all of the breast tumour cell lines tested (Figure 2B, *black bars*). This was confirmed to be apoptosis by annexin-V staining and through the use of caspase inhibitors that restored cell viability in a cell dependent manner (Additional file 1 Figure S4).

When c-FLIP inhibition (FLIPi) was combined with TRAIL administration, a significant TRAIL-dependent kill was observed for all of the breast cancer cell lines tested (Figure 2B, *white bars; *Additional file 1 Figure S5), demonstrating a marked sensitization to TRAIL in resistant cell lines, but no more than an additive effect of FLIPi in the TRAIL-sensitive MDA-MB-231 cell line. Thus, TRAIL/FLIPi had a marked effect on breast cancer cell viability irrespective of hormone receptor status. Despite the significant sensitization to TRAIL, between 8% (MDA-MB-231) and 33% (MCF-7) of the cell populations survived the combined (TRAIL/FLIPi) treatment (Figure 2C), which suggested a differential response to this apoptotic insult by these heterogeneous cell populations.

### FLIPi sensitized breast cancer stem cells (bCSCs) to TRAIL

Breast tumours and breast cancer cell lines, contain a small sub-population (up to 2%) of tumour initiating (cancer stem) cells [8]. These cells have been shown to be resistant to existing chemotherapeutic agents [47]. We wished to establish whether the cells surviving the TRAIL/FLIPi treatment within each cell line (Figure 2C) included a resistant sub-population of breast cancer stem cells (bCSCs).

**Figure 3:**
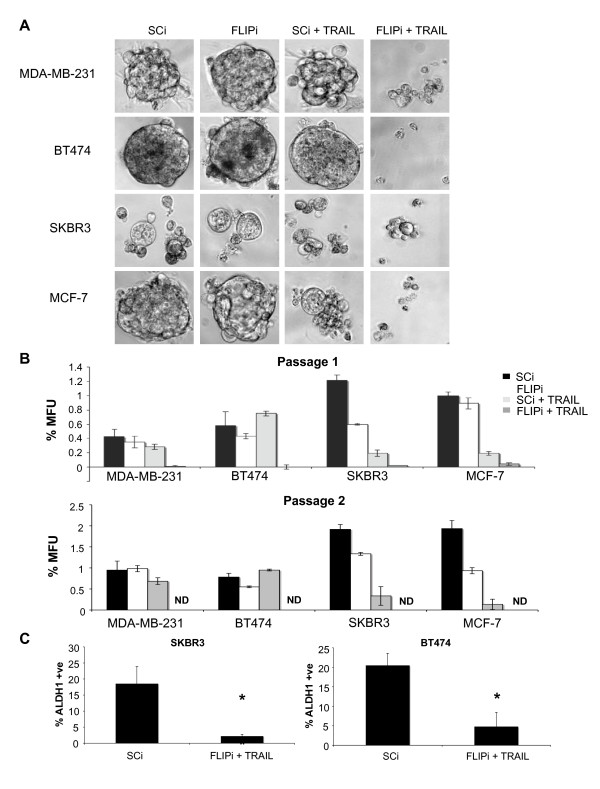
**FLIPi/TRAIL treatment inhibits mammosphere formation and prevents self-renewal of mammosphere forming units**. **A**, Following c-FLIP siRNAi (FLIPi) or control siRNA (SCi) transfection, cell lines were plated in low-serum non-adherent culture conditions in the presence or absence of 20 ng/ml TRAIL at a density of 4,000 cells per well (20,000 cells/ml). Pictures are representative of mammosphere forming units (MFU) observed. **B**, Mammospheres from three replicate wells per condition were counted following seven days culture (Passage 1). Mammospheres were dissociated using trypsin, passaged at a density of 2,000 cells/well (10,000 cells/ml) in the absence of TRAIL and counted after seven days culture (Passage 2). Results are calculated as a percentage of mammosphere forming units from the total number of cells seeded and are representative of three independent experiments. **C**, siRNA-transfected SKBR3 and BT474 cells were treated +/- 20 ng/ml TRAIL for 18 hours, surviving adherent monolayers were then assessed for aldefluor activity by flow cytometry. Graph represents the percentage of cells scoring positive for aldefluor activity in three independent experiments (* *P *< 0.01 vs. SCi control).

The proportion of bCSCs in each of the cell line's surviving cell population was determined using the functional mammosphere formation assay, as previously described [6,8,9]. Each of the cell lines was subjected to c-FLIP RNAi prior to transfer of viable cells to non-adherent conditions, whereupon cells were treated with TRAIL. Each of the untreated cell lines formed mammospheres of distinct size and morphology with the ER+ve lines, BT474 and MCF7, forming the largest, most uniform colonies (Figure 3A) and the ER-ve lines, SKBR3 and MDA-MB-231 forming loose, irregular colonies, as previously demonstrated [9]. Suppression of c-FLIP alone had no discernable effect on mammosphere integrity while TRAIL treatment alone partially impaired MCF-7 and MDA-MB-231 mammosphere morphology. Combined treatment, however, severely disrupted mammosphere formation in all four cell lines. This was confirmed by quantification of mammosphere forming units (MFUs) in short-term culture and serial passage (Figure 3B) whereby all self-renewing MFUs were deleted from the cell populations.

The frequency of mammosphere forming cells in the untreated cell lines ranged from 0.4% to 1.4% of the total cell populations. SKBR3 and MCF-7 MFUs were partially sensitive to TRAIL induced anoikis, as less than a quarter of the mammospheres formed in the presence of TRAIL alone during the first passage (Figure 3B, Passage 1). Similarly SKBR3, but not MCF-7, MFUs were significantly depleted with FLIPi treatment alone whilst MDA-MB-231 and BT474 cells were completely resistant to either FLIPi or TRAIL treatment alone. In all cases, however, sensitivity to anoikis was dramatically enhanced with combined treatment. From starting populations of 12,000 cells, no mammospheres survived in MDA-MB-231 and BT474 cultures, while two and one loose-forming colonies, respectively, were evident in SKBR3 and MCF-7 cells. Serial passaging of mammospheres in the absence of TRAIL and/or FLIPi revealed enrichment of MFUs in all cell cultures except those pre-treated with both TRAIL and FLIPi (Figure 3B). MFU enrichment is indicative of stem cell self-renewal due to symmetric cell division [8]. The complete loss of mammospheres from TRAIL/FLIPi treated cultures in subsequent passages suggests that the few surviving cancer initiating cells from 18 hours combined treatment were severely compromised and unable to undergo additional symmetric cell divisions. The same results were also observed using an alternative c-FLIP siRNA target sequence (Additional file 1 Figure S6).

The ablation of functional MFUs represents a preferential sensitization of bCSCs to TRAIL compared to the rest of the tumour cell population. This was confirmed by flow cytometry using the marker ALDH1 that has previously been shown to enrich HER2-positive breast cancer cell populations for tumour initiating cells (Additional file 1 Figure S7) [48]. The HER2-positive cell lines BT474 and SKBR3 were subjected to TRAIL/FLIPi or control siRNA (SCi) for 18 hours and only the surviving adherent cells stained for ALDH1 activity. Both cell lines exhibited significant reductions in the relative proportion of ALDH1 positive cells in the surviving populations following combined treatment (Figure 3C).

**Figure 4:**
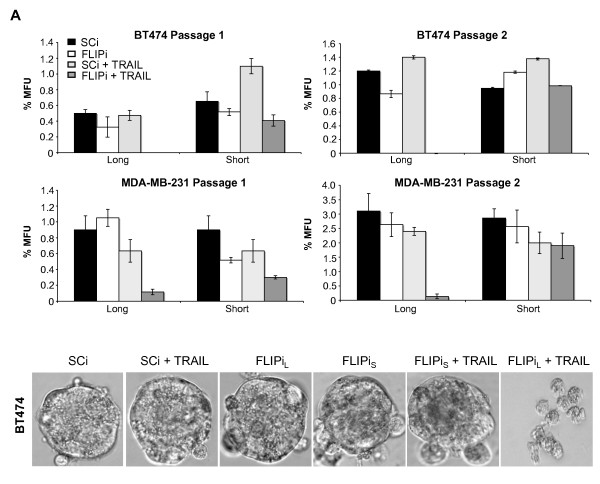
**c-FLIP_L _but not c-FLIP_S _knockdown sensitises breast cancer stem cells to TRAIL**. BT474 and MDA-MB-231 cells were transfected with siRNA sequences targeted specifically to the short or long c-FLIP isoforms (FLIPi) or control siRNA (SCi). Cells were then plated in mammosphere culture and treated with 20 ng/ml TRAIL.

**Figure 5:**
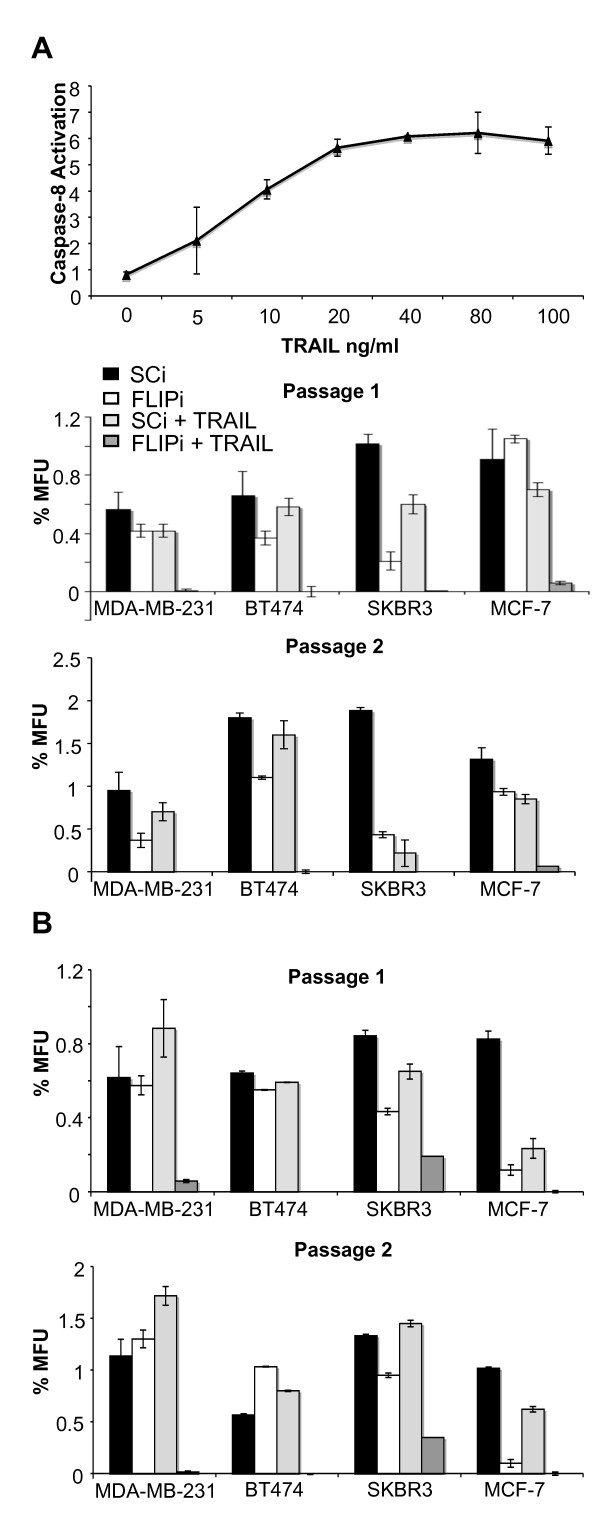
**Sensitization of cancer stem cells in adherent culture to TRAIL**. **A**, The TRAIL sensitive MDA-MB-231 cell line was subjected to increasing concentrations of TRAIL and in-well caspase-8 activity plotted as relative luminescence obtained by Caspase-Glo assay. Cell lines were transfected with siRNA (FLIPi and SCi) and plated in mammosphere conditions, as described in Figure 3, in the presence or absence of 1 ng/ml TRAIL. As in Figure 3, graphs represent the percentage of mammospheres (MFU) formed following seven days culture (Passage 1) and seven days after trypsinisation and re-seeding (Passage 2). **B**, Cell lines transfected with siRNA were treated with or without TRAIL for 18 hours while maintained in adherent monolayer culture. The surviving cell population was seeded at 20,000 cells/ml in low-serum, non-adherent culture conditions. Percentage mammosphere forming units (MFU) were calculated as previously described.

In order to address which c-FLIP isoform was responsible for the ablation of the self-renewing activity of the cancer stem cell population, siRNA sequences specific for cFLIP-short and c-FLIP-long transcripts were used prior to mammosphere assay. Silencing of c-FLIP-long, but not c-FLIP-short, mimicked the cytotoxic effects of global c-FLIP suppression in cancer stem cells, which confirmed an earlier observation of c-FLIP-long mediated survival in MCF-7 cells (Figure 4). Suppression of c-FLIP isoforms also sensitized cancer stem cells to sub-toxic levels of TRAIL (Figure 5). TRAIL concentrations were reduced from 20 ng/ml to 1 ng/ml, levels that failed to activate a cell death response in the TRAIL-sensitive MDA-MB-231 cell line (Figure 5A), and mammosphere cultures were performed as described above. TRAIL addition alone had reduced effects on mammosphere integrity, yet combined treatment abrogated MFUs in BT474, SKBR3 and MDA-MB-231 cell cultures, as previously observed with higher concentrations of TRAIL. The poorest responding cells to combined treatment, MCF-7 (Figure 3B), developed self-renewing MFUs at very low frequency (1/12,000 cells seeded) in reduced TRAIL conditions (Figure 5A).

The mammosphere formation assay primarily tests the cells' ability to resist anoikis, which is a key property of tumour initiating (cancer stem) cells. As c-FLIP has previously been reported to be an inhibitor of anoikis in other tumour cell types [49] we wished to test whether the MFU sensitization to TRAIL was dependent on the additional stresses imparted by the non-adherent conditions (Figure 5B). Each of the cell lines was subjected to c-FLIP RNAi and then incubated with TRAIL for 18 hours in adherent culture, as previously performed in the viability assays (Figure 1). Viable cells were subsequently washed and plated in non-adherent, mammospheres culture for seven days, in the absence of TRAIL, and the number of mammospheres counted. The self-renewing capacity of MFUs was once again abolished in MCF7, MDA-MB-231 and BT474 cell lines although SKBR3 cells exhibited a residual MFU self-renewal capacity. Thus while some cell lines exhibited reduced sensitivity to combined treatment when maintained in nurturing conditions, all continued to display a significant reduction in CSC properties.

### Tumour initiation and metastatic progression were compromised by combined TRAIL/cFLIPi treatment

**Figure 6:**
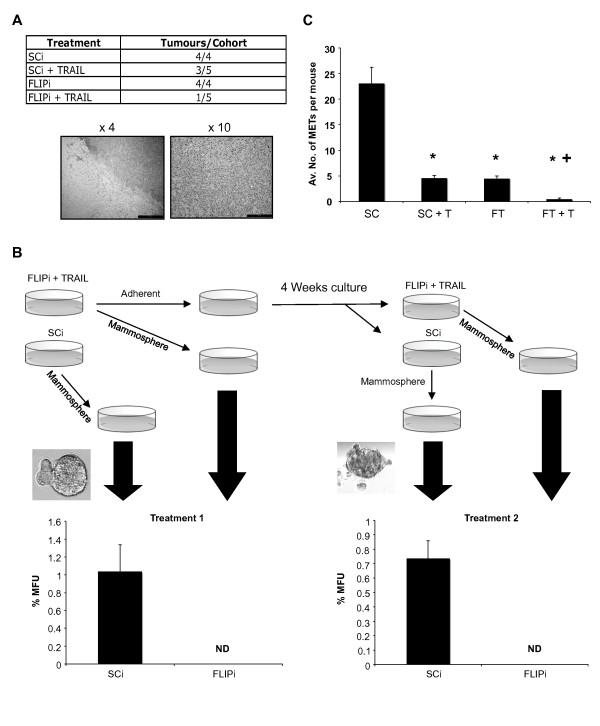
**FLIPi/TRAIL inhibits tumour initialisation and metastatic potential *in vivo***. **A**, BT474 cells were transfected with c-FLIP siRNA (FLIPi) or control siRNA (SCi) and 1 × 10^6 ^cells orthotopically transplanted in the presence or absence of 100 ng/ml TRAIL into the abdominal mammary fat pad of athymic nude mice in the presence of systemic estradiol. Transplant sites were monitored and measurements of palpable tumours taken twice weekly. **B**, FLIPi-transfected BT474 cells were treated for 18 hours with 20 ng/ml of TRAIL, the surviving adherent cells were then harvested and re-plated either in mammosphere culture (TRAIL/FLIPi Treatment 1) or adherent conditions at a density of 20,000 cells/ml. After four weeks culture, the adherent cell cultures were re-treated with FLIPi or SCi and 20 ng/ml TRAIL (18 hours) before plating for mammosphere assay (TRAIL/FLIPi Treatment 2). Images illustrate mammospheres formed in SCi conditions. **C**, 1 × 10^6 ^FLIPi (FT) or SCi (SC) treated MDA-MB-231 cells were injected, +/- 100 ng/ml TRAIL (T), into the tail veins of BALB/c severe combined immuno-deficient (SCID) mice. Mice were monitored daily and sacrificed six weeks post-surgery for histological examination of lung metastases.

In order to confirm that the loss of MFUs was consistent with a reduction in tumour initiating capacity, adherent cultures of BT474 cells were treated with c-FLIP siRNA and 10^6 ^viable cells orthotopically transplanted into the mammary glands of immune-compromised mice in the presence or absence of TRAIL (Figure 6A). The occurrence of palpable tumours was monitored for up to 16 weeks after transplantation. Tumours arose at the site of transplantation within eight weeks (Additional file 1 Figure S8A) of surgery in all mice transplanted with either untreated BT474 or FLIPi-treated BT474 cells, while three out of five mice with TRAIL-treated BT474 transplants acquired tumours in the same time-frame. However, four out of five transplants co-treated with FLIPi and TRAIL failed to acquire tumours within 16 weeks of surgery (Figure 6A). Tumour growth and histology were unaffected in all conditions.

This residual tumour initiating capacity following combined treatment occurred despite complete loss of self-renewing mammosphere forming potential *in vitro *(Additional file 1 Figure S8B). In order to determine whether this tumour initiating potential was re-acquired from the surviving (post-treatment) cell population, TRAIL/FLIPi treated cultures - with no residual mammosphere forming ability (Figure 6B, *Treatment 1*) - were maintained in adherent culture for four weeks then transferred to mammosphere culture or re-treated with TRAIL/FLIPi (Figure 6B, *Treatment 2*). The surviving population slowly re-populated the adherent conditions (Additional file 1 Figure S8C) and re-acquired an equivalent proportion of mammospheres to the original untreated population (compare SCi samples, Figure 6B, *Treatments 1 and 2*). However, this subset of self-renewing cells was still exquisitely sensitive to TRAIL/FLIPi, as combined treatment of the re-established adherent cultures once again eradicated MFUs from the cell population (Figure 6B, *Treatment 2*).

Cancer stem cells are thought to be responsible for the seeding of new tumour growth at distal sights, which is central to the progression of metastatic disease - the major cause of mortality in breast cancer patients. We used an established *in vivo *model of breast cancer metastasis, intravenous transplantation of MDA-MB-231 cells, to determine the effect of TRAIL/FLIPi on disease progression. Adherent cultures of MDA-MB-231 cells were treated with c-FLIP siRNA (or control) and 10^6 ^viable cells transplanted intravenously into immune-compromised mice in the presence or absence of TRAIL (Figure 6C). After six weeks the number of lung metastases was determined by dissection and serial section of lung tissues from recipient mice. An average of 23 secondary tumours per mouse were found in animals transplanted with control cells, compared to an average of 0.4 tumours (a total of two micrometastases from five mice) in transplants subjected to TRAIL and FLIPi. This represented a 98% reduction in tumour burden and a significant sensitization of TRAIL mediated suppression of metastatic disease (Figure 6C).

## Discussion

Tumour heterogeneity is a major obstacle to therapy. Recent insights into the hierarchical organisation of tumour cell populations highlights the potential importance of targeting the minority tumour-initiating (cancer stem) cell population associated with cancers in order to radically improve patient outcome. The problem is that cancer stem cells are inherently resistant to chemotherapeutic challenge.

Here we have shown, using complementary *in vitro *and *in vivo *functional assays, that inhibition of c-FLIP (FLIPi) overcomes resistance of breast cancer stem cells (bCSCs) to the anti-cancer agent TRAIL, resulting in the selective elimination of stem cell characteristics in all of the cell lines tested, independent of hormone receptor status. This potentially broadens the range of breast cancer subtypes that could benefit from a TRAIL-based therapy [18]. Formation of the DISC is a limiting factor in the initiation of the extrinsic apoptotic cascade [50,51]. We have confirmed that c-FLIP antagonises this cascade through the inhibition of either of the extrinsic initiator caspases, which cross-talk to the intrinsic pathway (caspase 9) [38]. The ability to de-repress either caspase-8 or -10 via FLIPi helps to explain the broad range of breast cancer cell types affected.

We found that combined TRAIL/FLIPi treatment *ex-vivo *had a marked impact on tumour seeding *in vivo*, resulting in a comprehensive suppression of lung metastases arising from circulating tumour cells (Figure 6). Significantly this occurred when TRAIL was co-injected with cells that had previously not been subjected to TRAIL while in cell culture. Despite this, however, a residual tumour initiating capacity persisted in the TRAIL/FLIPi cohort. This may be explained by our *in vitro *observations suggesting that bCSCs were marginally more resistant (Figure 5) and exhibited cellular plasticity (Figure 6B) in the nurturing microenvironment of adherent culture. The observation of functional plasticity in mammosphere culture supports a previous study using surrogate markers of bCSCs in breast cancer cell lines [52]. Crucially, however, we show that the newly acquired MFU activity remained responsive to re-administration of TRAIL/FLIPi. A similar sensitivity to repeat treatments has previously been observed for the Akt inhibitor perifosine, in a xenograft model of Sum159 cells [45]). These observations have important implications for the future prevention of disease relapse in the clinical setting as they demonstrate that the tumourigenic cell population may be targeted without selecting for resistant cells.

It has been suggested that tumour cells in their natural context do not necessarily exhibit the sensitivity to TRAIL monotherapy as observed *in vitro*, implying that a combined therapy would be required to re-sensitize to TRAIL [53]. We have used RNAi to demonstrate the proof of principle that suppression of c-FLIP expression in combination is sufficient to sensitize breast cancer cells to TRAIL. In light of this, a key future objective is to establish whether long-term suppression of c-FLIP *in vivo *- perhaps following the cessation of TRAIL treatment - might help prevent the recurrence of tumours. Despite limitations in drug design due to structural homology between c-FLIP and caspases, agents with broad specificity for c-FLIP have been described, each with anti-tumour properties [22,26,28,30,54,55]. It remains to be determined if these agents exhibit selective targeting of cancer stem cells and whether this is recapitulated *in vivo *in the absence of off target effects.

The breadth of the breast tumour cell types affected here raises the question of the potential ubiquity of FLIPi/TRAIL treatment in targeting other cancer types *in vivo*. Of the few studies that have addressed the sensitivity of cancer stem cells to TRAIL [16], the majority, including medulloblastoma [56], glioblastoma [19] and lymphoma [57]-derived stem cells, are resistant, with the exception of colorectal cancer cell lines in which a FACS sorted side-population was shown to be TRAIL responsive [58]. Sensitization of cancer stem cells to TRAIL has only previously been demonstrated in haematological cancers, including AML [55] and T-cell lymphoma cells [57], both of which have implicated, but not functionally proven, a role for c-FLIP in the process. TRAIL sensitization has not previously been described in solid tumour stem cells. Our study, therefore, is the first demonstration, to our knowledge, of TRAIL-mediated loss of functional stem cell activity in a solid tumour cell type and the first indication that CSC activity is directly influenced by c-FLIP.

Other mechanisms for targeting breast cancer stem cells have been described. Notably, a recent study demonstrated reduced stem cell activity in response to Notch1 or Notch4 suppression using the same breast cancer cell lines described herein [9], which supports the use of gamma-secretase inhibitors in clinical trials [47]. The Akt/Wnt pathway inhibitor, perifosine, reduces breast cancer stem cell numbers [45] and incidentally is responsible for the reduction in c-FLIP levels in AML stem cells [55]. Furthermore, it has been suggested that breast cancer stem cells may selectively express HER2 [59,45]) and that inhibition of this pathway could have beneficial consequences for breast cancer patients with both HER2-positive and HER2-negative disease [47,48]. As we have seen significant responses of CSCs to combined FLIPi/TRAIL, independent of HER2 receptor status, it will be of interest in the future to establish whether primary human tumour stem cell populations are equally susceptible and whether this is due to amplification of a DISC-related mechanism.

We have shown that the apoptosis observed following c-FLIP inhibition is, like TRAIL, a phenomenon that is relatively cancer-specific. Analysis of non-transformed mammary tissues from c-FLIP-deficient mice indicated that the absence of c-FLIP was not detrimental to normal tissue and did not sensitise normal tissue cells to TRAIL induced apoptosis. It has not been established, however, whether normal stem cells of the breast are affected by either intervention. Neural progenitor cells are resistant to TRAIL in a c-FLIP independent manner [60] and we are currently investigating whether murine mammary stem cells are similarly refractory.

## Conclusions

Taken together our results demonstrate that c-FLIP is a major inhibitor of TRAIL resistance in the tumour initiating (cancer stem) cell subset of a broad range of breast cancer cell lines. This work suggests that targeting c-FLIP may have important implications for the treatment of breast cancer in conjunction with TRAIL based therapeutics. Future studies are required, however, to address how cancer stem cells and normal stem cells residing in their respective niches *in vivo *would respond to FLIPi/TRAIL based therapy.

## Abbreviations

ALDH1: aldehyde dehydrogenase 1; bCSCs: breast cancer stem cells; c-FLIP: cellular FLICE-Like Inhibitory Protein; DISC: death inducing signalling complex; FLIPi: c-FLIP RNAi; HER2: heregulin receptor 2; MFU: mammosphere forming unit; SCi: scrambled control RNAi; TRAIL: tumour necrosis factor (TNF) Related Apoptosis Inducing Ligand.

## Competing interests

The authors declare that they have no competing interests.

## Authors' contributions

LP was responsible for the design of the experiments, assembly of data and manuscript writing. NO was responsible for the design of experiments, data collection and manuscript writing. SMP developed a new assay. ME handled data analysis and interpretation, funding of research and manuscript writing. RC was responsible for the conception and design of the study, data analysis and interpretation, manuscript writing, final approval of manuscript and funding of research. All authors have read and approved the manuscript for publication.

## Supplementary Material

Additional file 1 **Supplementary figures 1 to 8**. Figure S1. Western blot indicating parental cell line expression of epidermal growth factor receptor 2 (ErbB2) and estrogen receptor alpha (ERα). **Figure S2**. **A**, Representative flow cytometry plots and gating used to quantify dead cells from treatments described in the methods and **B**, FLIPi = c-FLIP siRNA. SCi = control siRNA. **Figure S3**. **A**, FLICE-Like Inhibitory Protein (c-FLIP) mRNA expression of viable cell population following transfection with siRNA. **B**, Western blot indicating relative c-FLIP protein expression of viable cells following treatment with c-FLIP siRNA (FLIPi) or control siRNA (SCi). **C**, Death receptor (DR)4 and DR5 expression in cell lines following c-FLIP suppression by siRNA. **Figure S4**. **A**, Cell lines were transfected with siRNA as previously described and apoptosis assessed by flow cytometry using Annexin-V staining (eBioscience). **B**, Cells were transfected with FLICE-Like Inhibitory Protein siRNA or scrambled control siRNA, in the absence (FLIPi) or presence of the caspase inhibitors IETD (caspase-8), LEHD (caspase-9) and AEVD (caspase-10) and apoptosis assessed by flow cytometry staining for Annexin-V. Results indicate the relative increase in Annexin-V staining of c-FLIP siRNA treated cells over their corresponding control siRNA. Cell death by FLIPi was either partially or completely inhibited by the IETD or AEVD demonstrating the cell death induced was a caspase-8 or caspase-10 dependant mechanism depending on cell line. LEHD also partially inhibited cell death in selected lines, confirming a previous report that c-FLIP induced activation of the extrinsic pathway impacted on the intrinsic apoptosis pathway. **Figure S5**. **A**, Representative phase contrast images of cell death analysed in Figure 2B. **B**, Cell lines were transfected with FLICE-Like Inhibitory Protein siRNA (FLIPi) or scrambled control siRNA (SCi) stained with PKH-26 (SCi) or PKH-67 (FLIPi), mixed at an equal ratio and treated with or without 20 ng/ml of Tumour Necrosis Factor-Related Apoptosis Inducing Ligand (TRAIL) for 18 hours. Cell death was then assessed by flow cytometry separating SCi and FLIPi cell death based on the membrane stain as in Figure 2B. **Figure S6**. Cells treated and quantified as in Figure 4 using an alternative set of oligonucleotides for FLICE-Like Inhibitory Protein (c-FLIP) targeted siRNA knockdown. **Figure S7**. Representative flow cytometry plots and gating used in the analysis of the aldefluor assay described in Figure 3C. **Figure S8. A**, Kaplen-Meier plot demonstrating time taken for first detection of tumour as described in Figure 5A. **B**, BT474 cells from Figure 5A were plated into mammosphere culture conditions at a density of 20,000 cells/ml and counted seven days later for percentage mammospheres (%MFU) calculation. **C**, Quantification of cell number over the four-week adherent culture period following Treatment 1 as described in Figure 5C. Click here for file
