# Erratum to: Suppression of apoptosis inhibitor c-FLIP selectively eliminates breast cancer stem cell activity in response to the anti-cancer agent, TRAIL

**DOI:** 10.1186/s13058-015-0597-9

**Published:** 2015-07-16

**Authors:** Luke Piggott, Nader Omidvar, Salvador Martí Pérez, Rhiannon French, Matthias Eberl, Richard WE Clarkson

**Affiliations:** Life Sciences Building, Cardiff University School of Biosciences, Museum Avenue, Cardiff, CF10 3AX Wales UK; Department of Infection, Immunity and Biochemistry, Cardiff University School of Medicine, Heath Park Campus, Cardiff, CF14 4XN Wales UK

## Correction

While compiling the article [1] one of the authors was inadvertently omitted from the author list. This author, Rhiannon French, has been included in the corrected author list above.
