# Supplementary figures and images for: Suppression of apoptosis inhibitor c-FLIP selectively eliminates breast cancer stem cell activity in response to the anti-cancer agent, TRAIL
Source: Breast Cancer Res. 2011 Sep 14;13(5):R88. doi: 10.1186/bcr2945 (PMC3262200; doi:10.1186/bcr2945)

Supp 1.

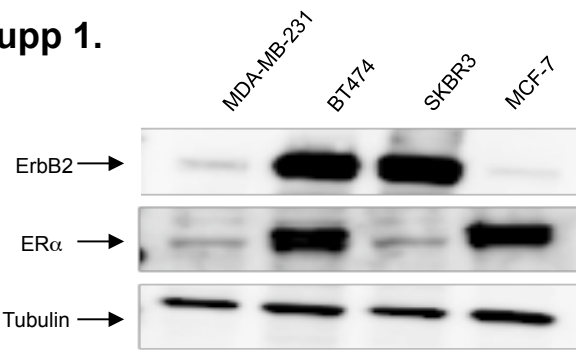

Supp 2.

A

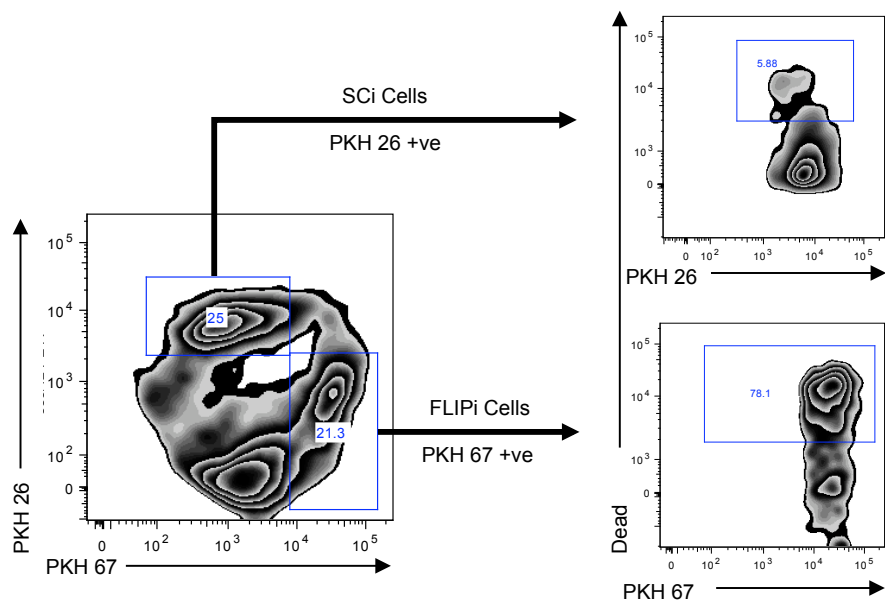

Supp 3.

A

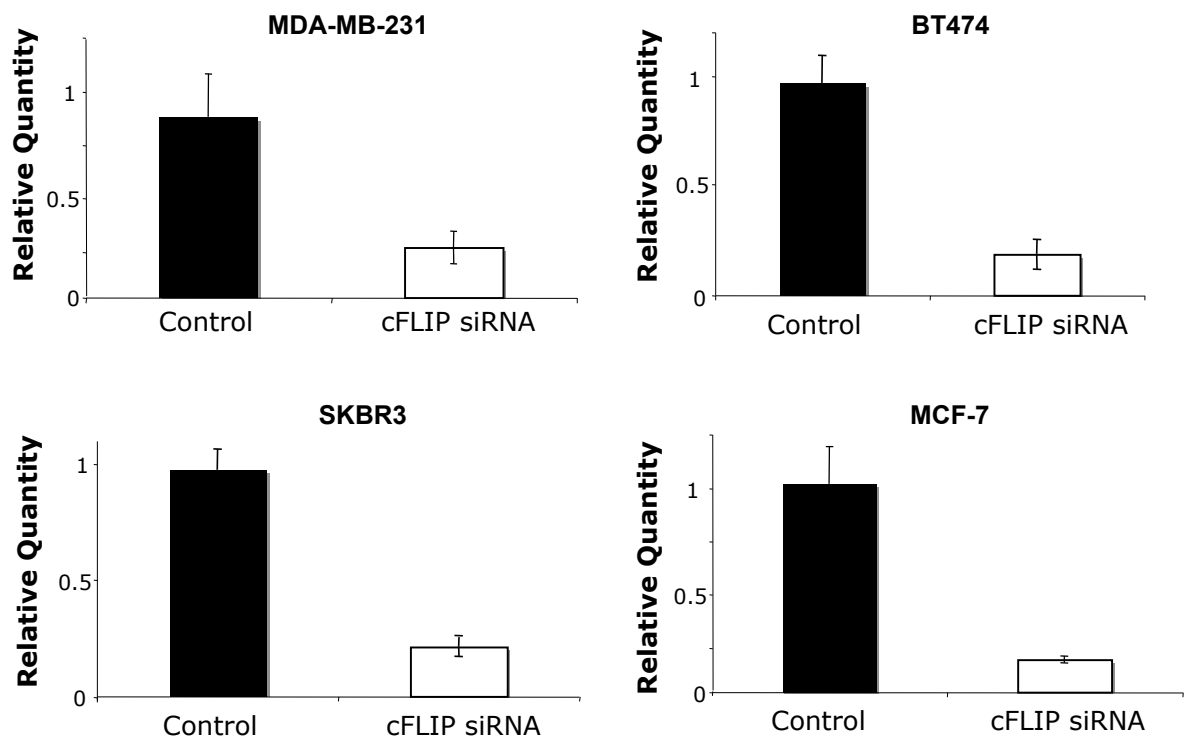

B

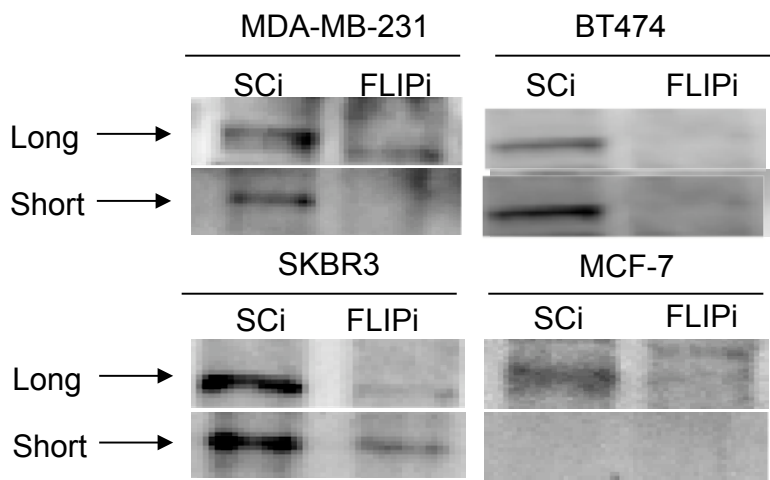

C

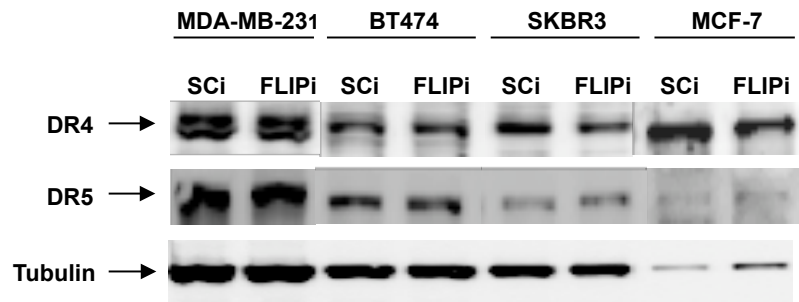

Supp. 4

A

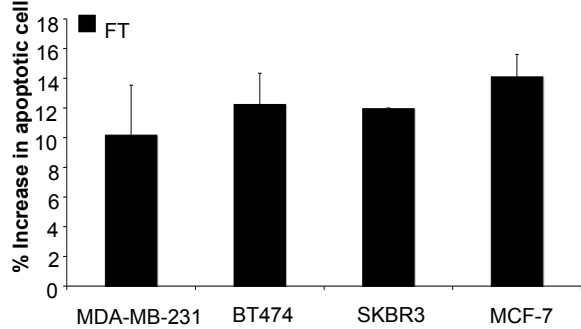

B

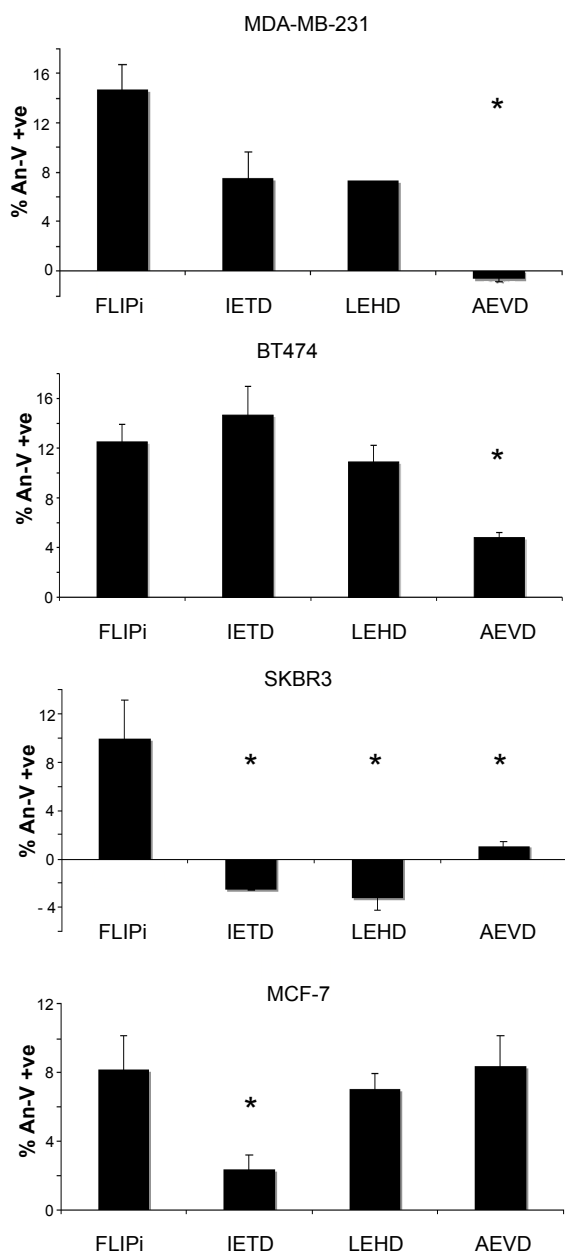

Supp 5.

A

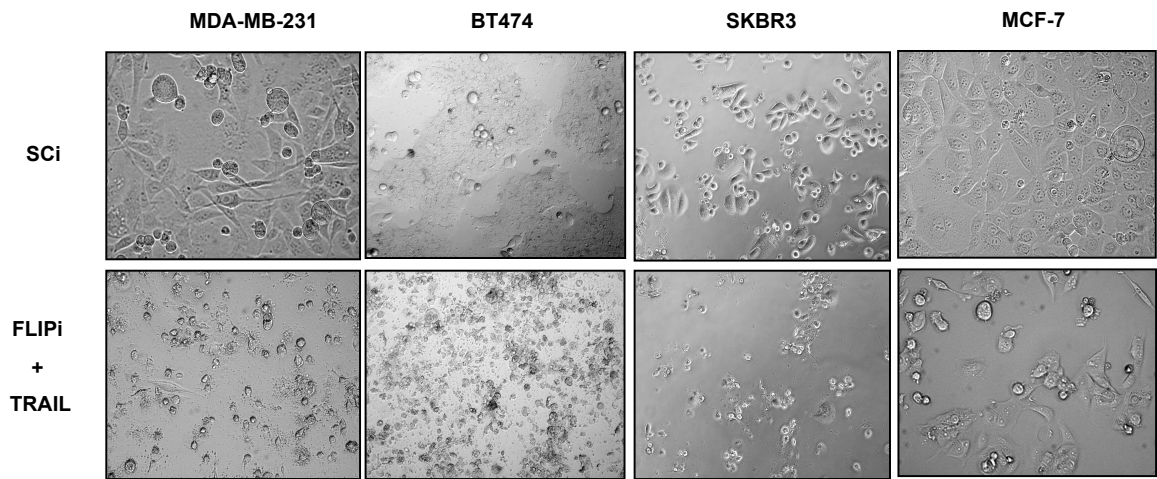

B

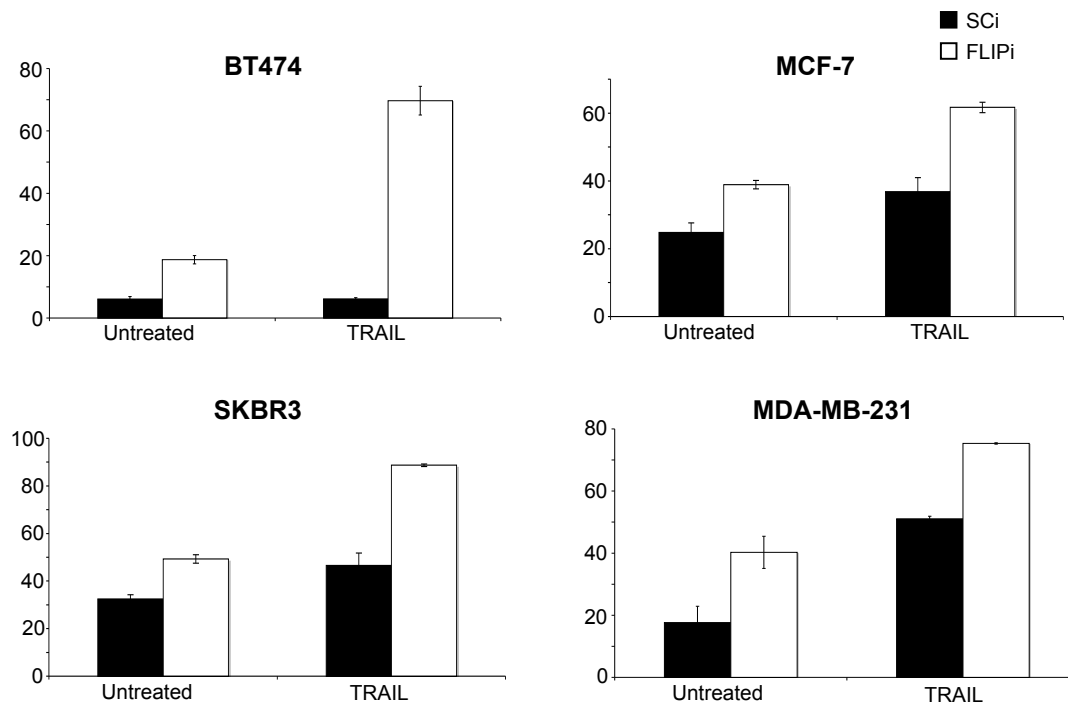

Supp 6.

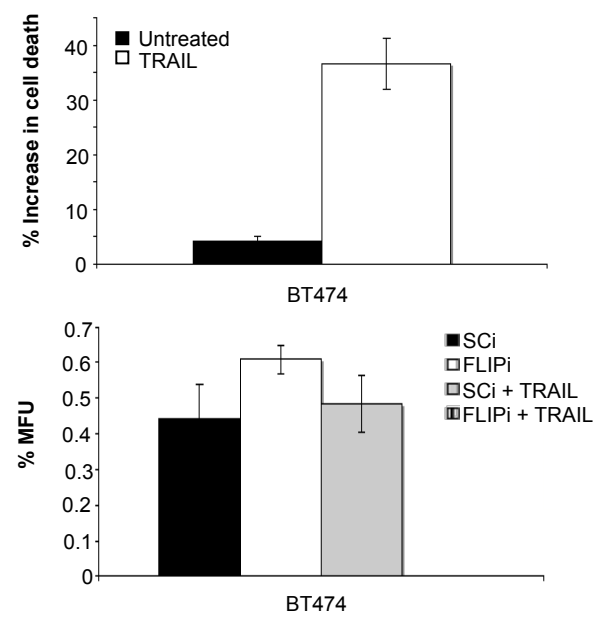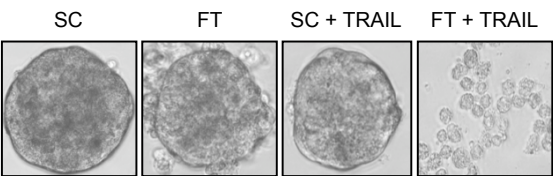

Supp 7.

SKBR3

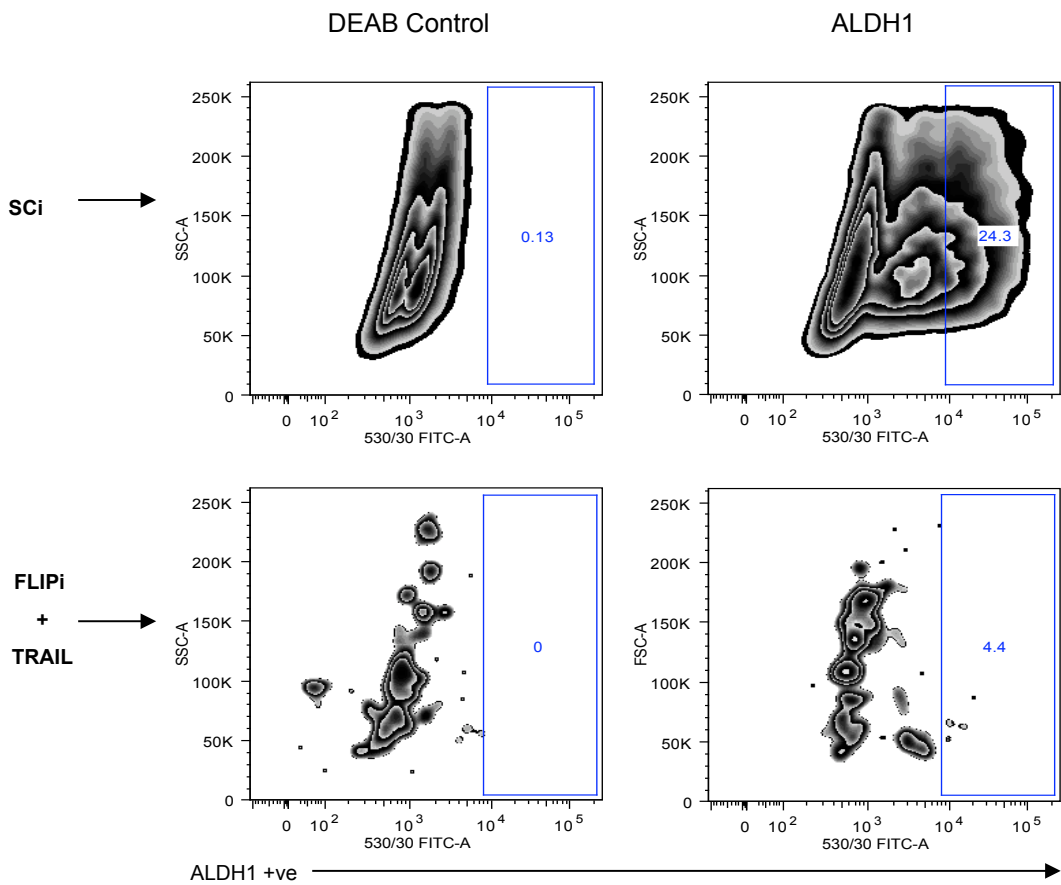

Supp 8.

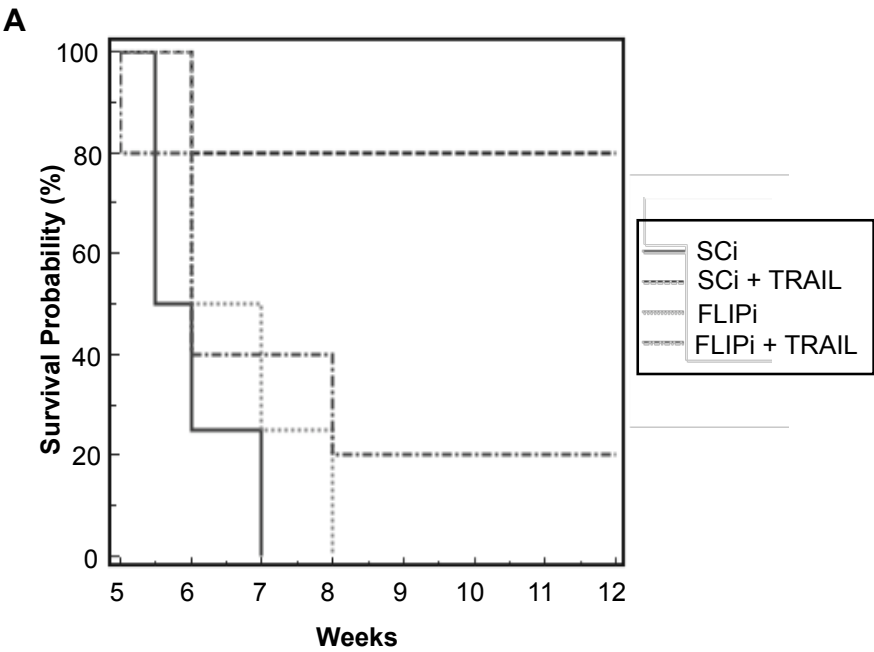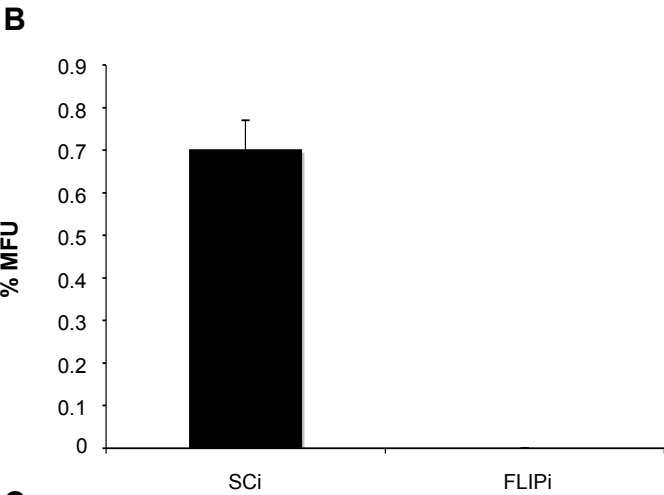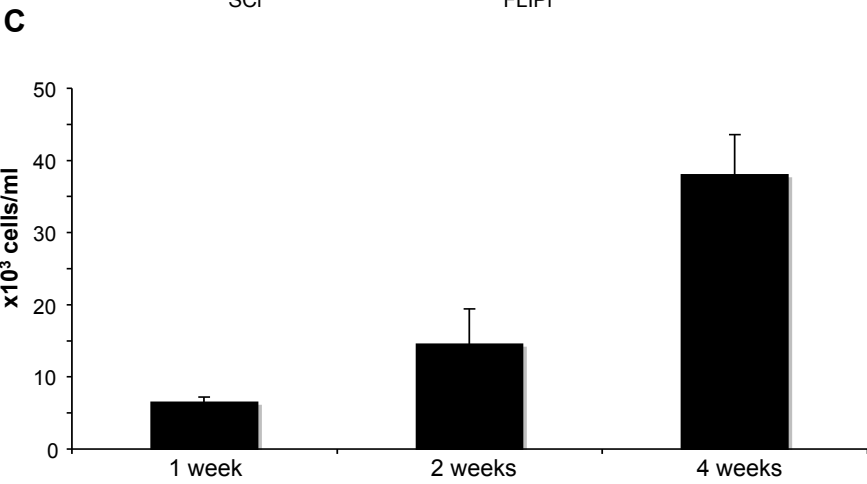

Supplement: Additional file 1 — Supplementary figures 1 to 8. Figure S1. Western blot indicating parental cell line expression of epidermal growth factor receptor 2 (ErbB2) and estrogen receptor alpha (ERα). Figure S2. A, Representative flow cytometry plots and gating used to quantify dead cells from treatments described in the methods and B, FLIPi = c-FLIP siRNA. SCi = control siRNA. Figure S3. A, FLICE-Like Inhibitory Protein (c-FLIP) mRNA expression of viable cell population following transfection with siRNA. B, Western blot indicating relative c-FLIP protein expression of viable cells following treatment with c-FLIP siRNA (FLIPi) or control siRNA (SCi). C, Death receptor (DR)4 and DR5 expression in cell lines following c-FLIP suppression by siRNA. Figure S4. A, Cell lines were transfected with siRNA as previously described and apoptosis assessed by flow cytometry using Annexin-V staining (eBioscience). B, Cells were transfected with FLICE-Like Inhibitory Protein siRNA or scrambled control siRNA, in the absence (FLIPi) or presence of the caspase inhibitors IETD (caspase-8), LEHD (caspase-9) and AEVD (caspase-10) and apoptosis assessed by flow cytometry staining for Annexin-V. Results indicate the relative increase in Annexin-V staining of c-FLIP siRNA treated cells over their corresponding control siRNA. Cell death by FLIPi was either partially or completely inhibited by the IETD or AEVD demonstrating the cell death induced was a caspase-8 or caspase-10 dependant mechanism depending on cell line. LEHD also partially inhibited cell death in selected lines, confirming a previous report that c-FLIP induced activation of the extrinsic pathway impacted on the intrinsic apoptosis pathway. Figure S5. A, Representative phase contrast images of cell death analysed in Figure 2B. B, Cell lines were transfected with FLICE-Like Inhibitory Protein siRNA (FLIPi) or scrambled control siRNA (SCi) stained with PKH-26 (SCi) or PKH-67 (FLIPi), mixed at an equal ratio and treated with or without 20 ng/ml of Tumour [file bcr2945-S1.PDF]
